# Effect of Sling Exercise Training on Balance in Patients with Stroke: A Meta-Analysis

**DOI:** 10.1371/journal.pone.0163351

**Published:** 2016-10-11

**Authors:** Lianghua Chen, Junqi Chen, Qiyuan Peng, Jingjie Chen, Yucong Zou, Gang Liu

**Affiliations:** Department of Rehabilitation medicine, The Third Affiliated Hospital of Southern Medical University, Guangzhou, Guangdong, China; University of South Australia, AUSTRALIA

## Abstract

**Objective:**

This study aims to evaluate the effect of sling exercise training (SET) on balance in patients with stroke.

**Methods:**

PubMed, Cochrane Library, Ovid LWW, CBM, CNKI, WanFang, and VIP databases were searched for randomized controlled trials of the effect of SET on balance in patients with stroke. The study design and participants were subjected to metrological analysis. Berg balance Scale (BBS), Barthel index score (BI), and Fugl-Meyer Assessment (FMA) were used as independent parameters for evaluating balance function, activities of daily living(ADL) and motor function after stroke respectively, and were subjected to meta-analysis by RevMan5.3 software.

**Results:**

Nine studies with 460 participants were analyzed. Results of meta-analysis showed that the SET treatment combined with conventional rehabilitation was superior to conventional rehabilitation treatments, with increased degrees of BBS (WMD = 3.81, 95% CI [0.15, 7.48], P = 0.04), BI (WMD = 12.98, 95% CI [8.39, 17.56], P < 0.00001), and FMA (SMD = 0.76, 95% CI [0.41, 1.11], P < 0.0001).

**Conclusion:**

Based on limited evidence from 9 trials, the SET treatment combined with conventional rehabilitation was superior to conventional rehabilitation treatments, with increased degrees of BBS, BI and FMA, So the SET treatment can improvement of balance function after stroke, but the interpretation of our findings is required to be made with caution due to limitations in included trials such as small sample sizes and the risk of bias. Therefore, more multi-center and large-sampled randomized controlled trials are needed to confirm its clinical applications.

## 1. Introduction

Stroke is a prevalent health problem and is one of the most common causes of acquired disability and death in adults[1, 2]. Patients suffering from stroke are prone to fall down due to balance dysfunction. The daily walking and activities of stroke patients are restricted[2, 3].Life quality be greatly impaired. In addition, confidence for patients returning their home life is affected. All these inconvenience resulting from stroke causes great burdens[4–6].

Common treatments for balance dysfunction include trunk muscle training exercises[1,2], mental imagery[7], functional electrical stimulation[8], motor relearning program[9], etc. Trunk muscle training exercises are the most commonly used clinical treatments because these activities strengthen core muscle functions and promotes recovery of balance function after stroke[10,11]. Intensive training on unstable support surfaces can enhance the stability of core muscles and enlarge the cross-sectional area of muscles, thereby increasing discharge frequency and numbers of motor unit. Therefore, unstable support training can be used to significantly improve balance than training on stable support surfaces[12–14].

Sling exercise training (SET)[15–17] has been recently emerged as a novel method of training trunk muscles on unstable supporting surfaces. This type of training has been demonstrated to activate trunk muscle activation based upon performance of active exercises with the help of sling exercise equipment. SET concerns the use of a dangling rope and auxiliary equipment to improve physical functions[18,19]. This type of training has been recently used to facilitate movement rehabilitation after stroke[18–26]. SET is a safe and partial body weight supporting training. SET could stimulate more proprioceptors, nerve roots, motor organs of the cerebrum and reactivate the muscles. Therefore, the SET could maximizing the sense of balance and enhancing trunk stabilization compared with traditional treatments[27,28].SET exhibits better therapeutic effects than traditional treatments, such as Bobath ball sports training and mat exercises[20–25,26].

However, several studies reported similar effect between SET and traditional treatments[18,19]. These contradicting findings were attributed to small sample sizes and limitations in the test design. The effect of SET on balance in patients with stroke must be further verified. Therefore, we performed a meta-analysis of randomized controlled studies (RCTs) on the efficacy of the SET treatment combined with conventional rehabilitation versus conventional rehabilitation treatment alone only on the recovery of balance function after stroke.

## 2. Materials and Methods

### 2.1 Literature Search Methods

We performed a systematic search of literature published prior to October 2, 2015 in PubMed, Cochrane Library, Ovid LWW, CBM (Chinese Biomedical), CNKI (China National Knowledge Infrastructure), WanFang, and VIP databases. The following search terms were used: stroke, strokes, stroke patient, cerebrovascular disorders, brain injuries, brain injury, chronic, paresis, hemiplegia, poststroke, infarction, thrombus, embolus cerebral, brain, haemorrhage, hemorrhage, haematoma, hematoma, bleed; suspension-assisted training, suspension-assisted, suspension, sling exercise, sling, sling exercise training; and balance, equilibrium, musculoskeletal equilibrium, musculoskeletal posture. Search was performed in accordance with the requirements for the database and search for the related literature.

### 2.2 Eligible Studies

#### (1) Inclusion criteria

**① Study design:** All RCTs on the effect of the SET treatment combined with conventional rehabilitation compared with conventional rehabilitation treatments published in Chinese or English were included in this analysis. The experimental group with the SET treatment with sling devices, device model, treatment of postures, movements, treatment time, intensity, frequency, and treatment courses were unlimited. Except for training trunk muscles, the conventional rehabilitation treatments of the experimental and control groups were unlimited.

**② Types of participants:** Patients with stroke manifesting balance dysfunction. Patients should meet the Fourth National Cerebrovascular Disease Conference diagnostic criteria in 1995[29] and the International Classification Of Diseases and Related Health Problems (ICD-10) cerebrovascular disease criteria (I60-I69). The diagnosis should have been confirmed through head CT or MRI diagnosis. Patients should be able to understand instructions.

**③ Types of outcome measures:** The following outcome measures should be included in the study:

1. evaluation index of balance: Berg Balance Scale (BBS)[30], sway area (SA) and sway length (SL)[18], BioRescue measures[19], and postural assessment scale for stroke patients (PASS)[31,32].
2. evaluation index of ambulatory capacity: Holden[33], functional ambulation category (FAC) [34], timed up and go test score (TUG)[18], and 10 m walking speed[24].
3. evaluation index of motor function: Fugl-Meyer Assessment (FMA)[35].
4. evaluation index of life ability and quality of life: Barthel index score (BI)[36], quality of life scale[37].
5. other evaluation indexes: Trunk Impairment Scale (TIS) [38], Frailty and Injuries Cooperative Studies of Intervention Technique (FICSIT-4) [39].

#### (2) Exclusion criteria

Studies were excluded if they did not conform to the inclusion criteria above, contained duplicate data, or were based on incomplete raw or irrelevant data. No case reports, letters, reviews, editorials, animal experimental studies, or correspondence articles were considered. In cases of multiple publications of the same or overlapping patients, only studies with the largest sample size were included.

### 2.3 Data Extraction

Two investigators (Chen Lianghua and Chen Jingjie) independently reviewed and extracted information from all eligible publications in accordance with the inclusion and exclusion criteria. Disagreement was resolved by discussion between the two authors. When a consensus was not reached, a third author (Peng Qiyuan) was consulted and a final decision was determined. Data extracted from the publications included the first author, year of publication, study design, number of participants (experimental/control group), interventions for both experimental and control groups (type and duration), outcome measures, the modified Jadad scale score, etc.

### 2.4 Methodological Quality

The methodological quality of each eligible study was independently assessed by two reviewers (Chen Lianghua and Chen Jingjie) based on the modified Jadad scale by using the following criteria[40]: (1) randomization procedure, (2) allocation concealment, (3) blinding procedure, and (4) drop-out explanation. Aggregate scores ranged from 0 to 7 points. Trials with scores between 4 and 7 were considered to have a higher methodological quality. Discrepancies between two independent evaluations for potential articles were resolved by Chen Junqi.

### 2.5 Statistical Analysis

Meta-analysis procedure was performed with the Review Manager 5.3. Results were expressed as odds ratio (OR) for dichotomous outcomes or weighted mean difference (WMD)/Standardized Mean Different (SMD) for continuous outcomes with 95% confidence intervals (CI).WMD is used when the units or measurement methods of the same intervention effect are identical. SMD is used when the effect of the same units or interventions are measured by different methods as well as the extremely large mean difference. Heterogeneity may be caused by the difference of intervention time, gender, geographical, the types of research, etc. We used the chi-square-based Q statistic (with a level of significance of P = 0.1) to evaluate the degree of heterogeneity between studies and quantified its extent with the *I*^*2*^ statistic. For P > 0.1 or *I*^*2*^ < 50%, the included studies were identified as having acceptable heterogeneity and the Fixed-effect model was used; otherwise, the random-effects model was used. Sensitivity analysis was performed and meta analysis was repeated to exclude abnormal results if the significant heterogeneity is available (P ≤ 0.1 and *I*^*2*^>50%). Then assess the stability of the integrative results between the two analysis. P value ≤ 0.1 was regarded as stable when comparing the two analysis.

## 3. Results

### 3.1 Literature Search

**Fig 1:**
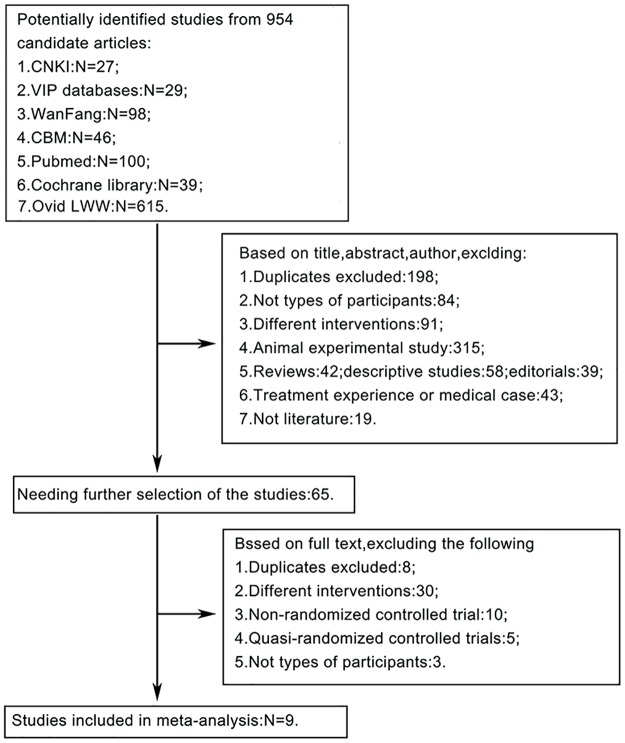
Flowchart showing the study selection procedure.

We initially retrieved 954 articles from the databases that were relevant to the search terms (Fig 1). Nine studies^[^^18^^–^^26^^]^ were finally included for analysis.

### 3.2 Study characteristics

**① Study design:** Nine studies[18–26] were included for randomized controlled trials.

**② Types of participants:** Of the nine studies, 460 patients with stroke were included. Seven studies diagnosed stroke using the Fourth National Cerebrovascular Disease Conference diagnostic criteria in 1995[27] and the International Classification of Diseases and Related Health Problems (ICD-10) cerebrovascular disease criteria (I60-I69). The participants had to be confirmed through head CT or MRI diagnoses and they should be able to understand instructions.

**Fig 2:**
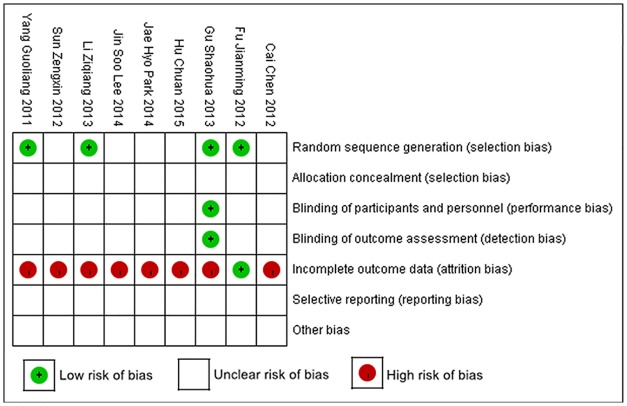
Risk of bias summary: review of the authors’ judgments about each risk of bias item for each included study.

**③ Characteristics of the included studies (Table 1) and risk of bias summary (Fig 2).**

**Table 1:** Characteristics of the studies included in the meta-analysis.

Author, year	Patient Characteristics, Sample Size	Intervention Onset period	Duration of trial period	Time point	Outcomes
Yang Guoliang, 2011[20]	Source:96 patients diagnosed with Stroke (G1 = 48, male: 26, female: 22; G2 = 48, male: 29, female: 19); Mean age (SD): G1 = 58.29 y (9.33); G2 = 58.91 y (8.67)	G1:conventional rehabilitation, 6.59 d (3.31); G2:SET combined with conventional rehabilitation, 6.27 d (3.08)	two times per day for 8 weeks, 45 min every time	8weeks	1.BI; 2.FMA (Lower limb); 3. 6m walking speed; 4.PASS
Sun zengxin, 2012[21]	Source: 40 patients diagnosed with Stroke (G1 = 20, male: unclear, female: unclear; G2 = 20, male: unclear, female: unclear); Mean age (SD): G1 = 58.29 y (9.33); G2 = 58.91 y (8.67)	G1:conventional rehabilitation, 8.95m (2.038); G2:SET, 9.25 m (1.86)	0.5 min five times a week for 2 weeks	2weeks	1. 10m walking speed; 2.Extending forward from the upper limbs; 3.standing time of one suffering leg; 4.TIS
Cai chen, 2012[22]	Source: 80 patients diagnosed with Stroke (G1 = 40, male: 26, female: 14; G2 = 40 male: 28, female: 12; Mean age (SD): G1 = 64.3 y (2.4); G2 = 62.8 y(4.7)	G1:conventional rehabilitation,12.51 d (2.4); G2:SET combined with conventional rehabilitation, 12.24 d (2.7)	two times per day, 6 days a week for 8 weeks, 40 min every time	8 weeks	1.FMA (Lower limb); 2. 10 m walking speed; 3.Holden
Fu jianming, 2012[23]	Source: 20 patients diagnosed with Stroke (G1 = 10, male: 7, female: 3; G2 = 10, male: 5, female: 5; Mean age (SD): G1 = 61.1 y (4.9); G2 = 60.5 y(5.2).	G1: conventional rehabilitation, 99.0 d (6.2); G2: SET combined with conventional rehabilitation, 98.0 d (5.8).	0.5 hour five times a week for 8 weeks.	8 weeks	1.BBS; 2.FMA
Gu shaohua, 2013[24]	Source: 24 patients diagnosed with Stroke (G1 = 12, male: 7, female: 5; G2 = 12, male: 9, female: 3; Mean age(SD): G1 = 58.8 y (8.4); G2 = 60.3 y(7.9).	G1:conventional rehabilitation, 52.9 d (7.8); G2:SET combined with conventional rehabilitation, 55.8 d (8.1)	0.5 hour class five times a week for 4 weeks.	4 weeks	1.BBS; 2.FMA (Lower limb); 3. 10 m walking speed; 4. FAC
Li ziqiang 2013[25]	Source: 80 patients diagnosed with Stroke (G1 = 40, male: 24, female: 16; G2 = 40, male: 23, female: 17; Mean age (SD): G1 = 58.00 y (17.52); G2 = 60.00 y (16.97).	G1:conventional rehabilitation, unclear; G2:SET combined with conventional rehabilitation, unclear	Two times per day for 2 weeks, 50 min every time.	2 weeks	1.BBS; 2.FMA; 3.BI.
Park,J.H.2014[18]	Source: 40 patients diagnosed with Stroke (G1 = 20, male: 13, female: 7; G2 = 20, male: 11, female: 9; Mean age (SD): G1 = 48.65 y (12.81); G2 = 51.15 y (14.81).	G1: conventional rehabilitation, 12.75 m (9.6); G2: SET, 14.10 m (11.40)	30 min three times a week for 8 weeks.	8 weeks	1.SA; 2.SL
Lee, J.S. 2014[19]	Source: 20 patients diagnosed with Stroke (G1 = 10, male: unclear, female: unclear; G2 = 10, male: unclear, female: unclear; Mean age (SD): G1 = 62.50 y (8.48); G2 = 63.40 y (4.94)	G1:conventional rehabilitation, more than 24 m; G2:SET, more than 24 m	30 min three times a week for 4 weeks	4 weeks	1.BBS; 2.FICSIT-4; 3.TUG; 4.BioRescue score; 5.EMG
Hu chuan, 2015[26]	Source: 60 patients diagnosed with Stroke (G1 = 30, male: 21, female: 9; G2 = 30, male: 22, female: 8; Mean age (SD): G1 = 59.13 y (9.23); G2 = 58.36 y (10.41)	G1:conventional rehabilitation, 3.63m (1.17); G2: SET combined with conventional rehabilitation, 3.92m (1.04)	30 min six times a week for 4 weeks	4 weeks	1.BBS; 2.FMA (Lower limb)

**Abbreviations** G: Group; G1 = control group; G2 = experimental group; SD: Standard deviation; y: year; m: month; BBS: Berg Balance scale; FMA: Fugl–Meyer Assessment; EMG: Surface electromyography; TUG: Timed Up & Go; FICSIT-4: Frailty and Injuries Cooperative Studies of Intervention Technique; SA: sway area; SL: sway length; BI: Barthel Index; FAC: functional ambulation category; Holden: Holden walking function classification; TIS: Trunk impairment scale; PASS: the Postural Assessment Scale for stroke patients.

Table 1 shows that each of the included studies are referred to by a random method. However, specific methods of generating random sequence were not described in detail, and sample size trials were small. Table 1 also shows that each of the included studies Characteristics, included Patient Characteristics, Sample Size, Intervention Onset period, Duration of trial period, Time point, Outcomes. The subjects included 460 patients from 9 studies diagnosed with stroke. Rehabilitation training began 48 hours to over 24 months after the stability phase of cerebral stroke. The parameters for evaluating studies Characteristics were different, included timing post stroke and amount of disability. The BBS, BI, and FMA were the most common types of outcome measures.

### 3.3 Assessment of BBS

**Fig 3:**
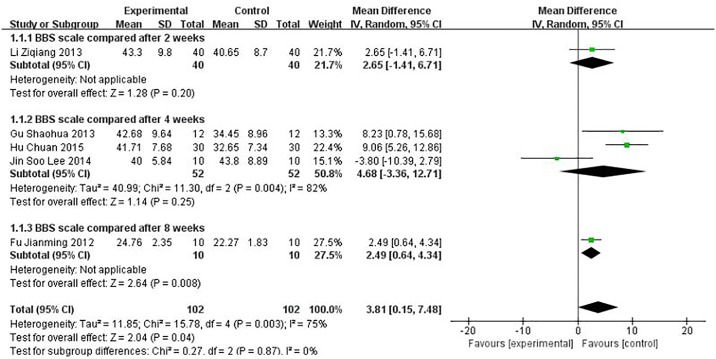
Effect of sling exercise training (SET) compared with conventional rehabilitation alone for BBS on forest plot.

**Fig 4:**
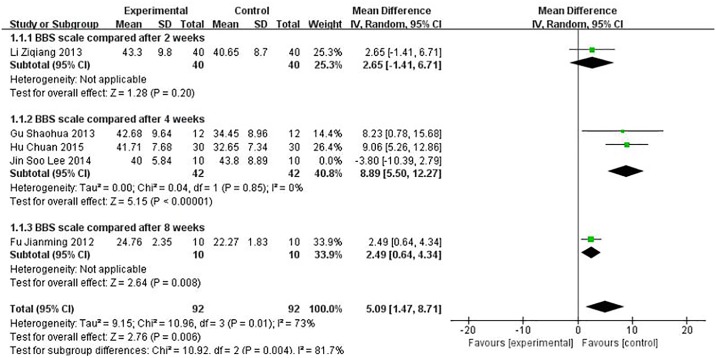
Sensitivity analysis effect of sling exercise training (SET) compared with conventional rehabilitation alone for BBS.

Five studies using BBS were performed to evaluate the effect of the treatment. The BBS score for the meta-analysis of SET was significantly improved (WMD = 3.81, 95% CI [0.15, 7.48], P = 0.04) compared with conventional rehabilitation alone, as shown in Fig 3 (P ≤ 0.1 and I2 = 75%); thus, the heterogeneity existed in every study. We further conducted sensitivity analysis, and the results were stable, as shown in Fig 4.

### 3.4 Assessment of BI

**Fig 5:**
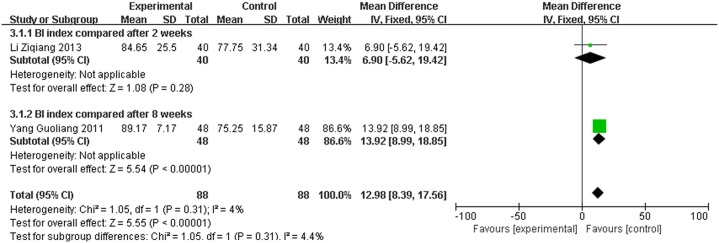
Effect of sling exercise training (SET) compared with conventional rehabilitation alone for BI on forest plot.

Two studies used BI to evaluate the effect of the treatment. The BI score for the meta-analysis of SET was significantly improved (WMD = 12.98, 95% CI [8.39, 17.56], P < 0.00001) compared with conventional rehabilitation alone, as shown in Fig 5.

### 3.5 Assessment of FMA

**Fig 6:**
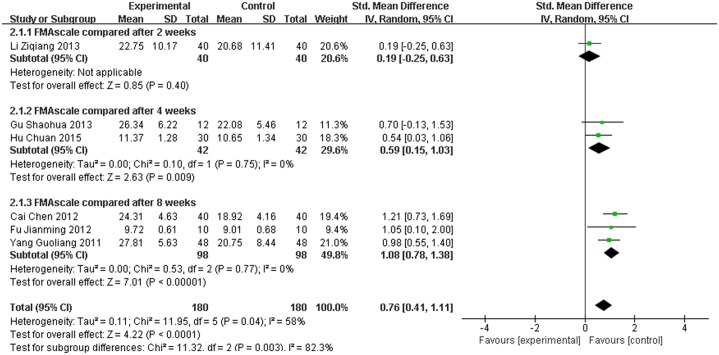
Effect of sling exercise training (SET) compared with conventional rehabilitation alone for FMA on forest plot.

**Fig 7:**
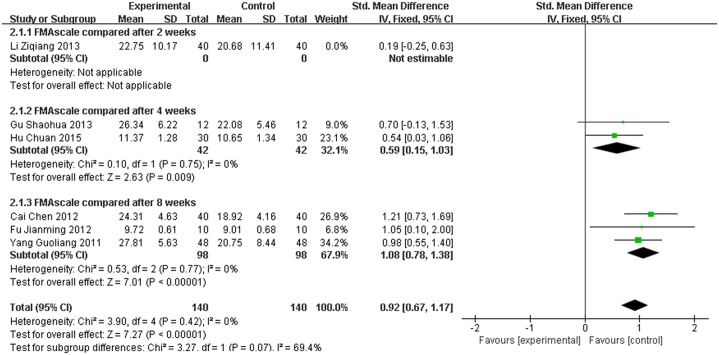
Sensitivity analysis results of sling exercise training (SET) compared with conventional rehabilitation alone for FMA.

Six studies used FMA to evaluate the effect of the treatment. The FMA score for the meta-analysis of SET was significantly improved (SMD = 0.76, 95% CI [0.41,1.11], P < 0.0001) compared with conventional rehabilitation alone, as shown in Fig 6. However, P ≤ 0.1 and I2 = 58%; thus, heterogeneity existed in every study. More sensitivity analyses were conducted, and the results were stable, as shown in Fig 7.

### 3.6 Other Types of Outcome Measures

A combined meta-analysis cannot be performed because of the limited number of included studies and different evaluation methods employed. As such, we performed descriptive analysis Yang Guoliang et al.[20] showed that 6 m walking speed and PASS significantly improved after SET. Sun zengxin et al.[21] showed that TIS, 10 m walking speed, extending forward from the upper limbs, and standing time of one suffering leg were significantly improved after SET. Cai chen et al.[22] showed that 10 m walking speed and Holden were significantly improved after SET. Gu shaohua et al.[24] showed that 10 m walking speed and FAC significantly changed after SET. Park, J.H.et al.[18] showed that BioRescue measures such as sway area (SA) and sway length (SL) significantly changed after SET and were similar to those of maximum training performed on the unstable support surface. Lee, J.S. et al.[19] showed that FICSIT-4, TUG, BioRescue score, and EMG significantly changed after SET but were not different from those of maximum training on the unstable support surface.

## 4. Discussion

This review is the first systematic meta-analysis that evaluated the effect of SET on balance in patients with stroke. We included nine trials with a total of 460 patients and found that SET combined with conventional rehabilitation may significantly improve movement after stroke compared with conventional rehabilitation in terms of BBS, BI and FMA scores. In conclusion, some evidence may prove that SET may affect movement after stroke, especially balance.

BBS, BI, and FMA are commonly used to evaluate balance dysfunction, ADL and motor function after stroke respectively. In trials of our manuscript, Patients with stroke manifesting balance dysfunction were included, Improvement of ADL and motor function can evaluate the balance function of indirect reaction. The trend of BBS, BI, and FMA scores fluctuation were consistent. Balance in parallel with changes in BI and FMA scores. So BBS, BI, and FMA scores used to evaluate balance dysfunction in this article. We found that SET improved balance after stroke by increasing these subjective indicator scales, supporting the findings of Lee and Park[18,19]. They found that surface EMG was used to measure trunk muscle activity during the interventions, and the Biofeedback analysis system of BioRescue was used to measure the moving distance, area, and speed of the participant’s center of gravity with weights placed on both legs. The result showed that SET can increase trunk muscle activity and decrease the moving distance, area, and speed. This study provides an objective basis for SET to improve balance dysfunction after stroke.

SET using fixed sling can provide a stable support surface for static balance training, but its length, height and elasticity can also be adjusted for dynamic balance training. Adjustment can be done by setting different moving points and sling power to increase the difficulty of movement, trunk muscle strength, and endurance; moreover, coordination in balance training can be based on the overall improvement of trunk muscle function and increased proprioceptive input, thereby promoting posture control and balance after stroke[41].

The difference resulted in large heterogeneity among each study, such as observation time points, patients with motor dysfunction, etc. The forest plot can be used to identify publication bias and heterogeneous size effect. According to forest plot results, we canceled forest plot foreign literature and sensitivity analysis, and found that the results of meta-analyses were stable.

In summary, SET significantly improved balance function in patients after stroke compared with conventional rehabilitation alone. We recommend the use of SET to regain balance function after stroke. SET increases the ability to control the trunk and balance on unstable support surfaces, thereby promoting patients return to their family and society early.

However, the present meta-analysis has some limitations. We included nine studies and the test methods described were different. The varied randomization procedures were not perfect, parts of tests did not specify the kinds of randomization methods, and SET is difficult blinding of patients and doctors, resulting in bias. These may affect test results and high-quality filter paper documents are difficult to obtain. The included studies cover only small sample sizes. Moreover, most trials did not provide drop-out explanations and only partial follow-up tests were carried out to assess the therapeutic effect. Most studies found no consistent effect score, and evaluation was also different, making it is difficult to generalize and obtain authentic meta-analysis results. To obtain more accurate results, larger samples are needed for the multi-center prospective trials and high-quality randomized trials.

## 5. Conclusions

Based on limited evidence from 9 trials, the SET treatment combined with conventional rehabilitation was superior to conventional rehabilitation treatments, with increased degrees of BBS, BI and FMA, So the SET treatment can improvement of balance function after stroke, but the interpretation of our findings is required to be made with caution due to limitations in included trials such as small sample sizes and the risk of bias. Therefore, more multi-center and large-sampled randomized controlled trials are needed to confirm its clinical applications.

## Supporting Information

S1 File PRISMA flow diagram. Flow diagram showing the study selection procedure.
(DOC)

S2 File PRISMA checklist. Checklist showing the main contents of this paper and the corresponding position.
(DOC)
