# Supplementary material for: Effect of Sling Exercise Training on Balance in Patients with Stroke: A Meta-Analysis
Source: PLoS One. 2016 Oct 11;11(10):e0163351. doi: 10.1371/journal.pone.0163351 (PMC5058486; doi:10.1371/journal.pone.0163351)
Supplement: S1 File — Flow diagram showing the study selection procedure. (DOC) [file pone.0163351.s001.doc]

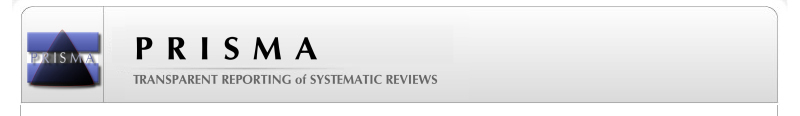
**PRISMA Flow Diagram**

Records identified through database articles in Chinese/English

clinical trials searching
CNKI (n =27)

VIP databases (n =29)

Wanfang (n =98)

CBM (n =46)

Pubmed (n =100)

Cochrane library (n =39)

Ovid LWW (n =615)

**Screening**

**Included**

**Eligibility**

**Identification**

Additional records identified through other sources
(n = 0)

Records after duplicates removed
(n = 954 )

Records screened
(n =756)

Records excluded:

1. Duplicates excluded (n =198 )

2. Not types of participants (n = 84)

3. Different interventions (n =91)

4. Animal experimental study (n = 315)

5. Reviews (n =42); descriptive studies(n =58); editorials(n =39)

6. Treatment experience or medical case (n =43)

7. Not literature(n =19)

Full-text articles assessed for eligibility
(n =65)

Full-text articles excluded, with reasons:

1. Duplicates excluded(n =8)

2. Different interventions(n =30)

3. Non-randomized controlled trial(n =10)

4. Quasi-randomized controlled trials(n =5)

5. Not types of participants(n =3)

Studies included in qualitative synthesis
(n =9)

Studies included in quantitative synthesis (meta-analysis)
(n =9)
