# Supplementary material for: Effect of Sling Exercise Training on Balance in Patients with Stroke: A Meta-Analysis
Source: PLoS One. 2016 Oct 11;11(10):e0163351. doi: 10.1371/journal.pone.0163351 (PMC5058486; doi:10.1371/journal.pone.0163351)
Supplement: S2 File — Checklist showing the main contents of this paper and the corresponding position. (DOC) [file pone.0163351.s002.doc]

| **Section/topic** | **#** | **Checklist item** | **Reported on page #** |
| --- | --- | --- | --- |
| **TITLE** | | |  |
| Title | 1 | Effect of sling exercise training on balance in patients with stroke: A meta-analysis. | Manuscript1 |
| **ABSTRACT** | | |  |
| Structured summary | 2 | Background  Stroke is a prevalent health problem and is one of the most common causes of acquired disability and death in adults. Patients suffering from stroke are prone to fall down due to balance dysfunction. The daily walking and activities of stroke patients are restricted. Life quality be greatly impaired . In addition, confidence for patients returning their home life is affected. All these inconvenience resulting from stroke causes great burdens.  Common treatments for balance dysfunction include trunk muscle training exercises, mental imagery, functional electrical stimulation, motor relearning program, etc. Trunk muscle training exercises are the most commonly used clinical treatments because these activities strengthen core muscle functions and promotes recovery of balance function after stroke. Intensive training on unstable support surfaces can enhance the stability of core muscles and enlarge the cross-sectional area of muscles, thereby increasing discharge frequency and numbers of motor unit. Therefore, unstable support training can be used to significantly improve balance than training on stable support surfaces.  Sling exercise training (SET) has been recently emerged as a novel method of training trunk muscles on unstable supporting surfaces. This type of training has been demonstrated to activate trunk muscle activation based upon performance of active exercises with the help of sling exercise equipment. SET concerns the use of a dangling rope and auxiliary equipment to improve physical functions. This type of training has been recently used to facilitate movement rehabilitation after stroke. SET is a safe and partial body weight supporting training. SET could stimulate more proprioceptors , nerve roots, motor organs of the cerebrum and reactivate the muscles. Therefore, the SET could maximizing the sense of balance and enhancing trunk stabilization compared with traditional treatments. SET exhibits better therapeutic effects than traditional treatments, such as Bobath ball sports training and mat exercises.  Objectives  This study aims to evaluate the effect of sling exercise training (SET) on balance in patients with stroke.  Data sources  PubMed, Cochrane Library, Ovid LWW, CBM, CNKI, WanFang, and VIP databases.  Study eligibility criteria  All RCTs on the effect of the SET treatment compared with conventional rehabilitation treatments published in Chinese or English were included in this analysis.  Participants  Patients with stroke manifesting balance dysfunction. Patients should meet the Fourth National Cerebrovascular Disease Conference diagnostic criteria in 1995 and the International Classification Of Diseases and Related Health Problems (ICD-10) cerebrovascular disease criteria (I60-I69). The diagnosis should have been confirmed through head CT or MRI diagnosis. Patients should be able to understand instructions.  Interventions  The experimental group with the SET treatment with sling devices, device model, treatment of postures, movements, treatment time, intensity, frequency, and treatment courses were unlimited. Except for training trunk muscles, the conventional rehabilitation treatments of the experimental and control groupswere unlimited.  Study appraisal and synthesis methods  Berg balance Scale (BBS), Barthel index score (BI), and Fugl-Meyer Assessment (FMA) were used as independent parameters for evaluating balance function , activities of daily living(ADL) and motor function after stroke respectively, and were subjected to meta-analysis by RevMan5.3 software.  Results  Results of meta-analysis showed that the SET treatment combined with conventional rehabilitation was superior to conventional rehabilitation treatments, with increased degrees of BBS (WMD = 3.81, 95% CI [0.15, 7.48], P = 0.04), BI (WMD = 12.98, 95% CI [8.39, 17.56], P < 0.00001), and FMA (SMD =0.76, 95% CI [0.41, 1.11], P < 0.0001).  Limitations  However, the present meta-analysis has some limitations. We included nine studies and the test methods described were different. The varied randomization procedures were not perfect, parts of tests did not specify the kinds of randomization methods, and SET is difficult blinding of patients and doctors, resulting in bias. These may affect test results and high-quality filter paper documents are difficult to obtain. The included studies cover only small sample sizes. Moreover, most trials did not provide drop-out explanations and only partial follow-up tests were carried out to assess the therapeutic effect. Most studies found no consistent effect score, and evaluation was also different, making it is difficult to generalize and obtain authentic meta-analysis results. To obtain more accurate results, larger samples are needed for the multi-center prospective trials and high-quality randomized trials.  Conclusions  Based on limited evidence from 9 trials, the SET treatment combined with conventional rehabilitation was superior to conventional rehabilitation treatments, with increased degrees of BBS, BI and FMA, So the SET treatment can improvement of balance function after stroke, but the interpretation of our findings is required to be made with caution due to limitations in included trials such as small sample sizes and the risk of bias. Therefore, more multi-center and large-sampled randomized controlled trials are needed to confirm its clinical applications. | Manuscript 1/2/3/4/6 |
| **INTRODUCTION** | | |  |
| Rationale | 3 | Trunk muscle training exercises are the most commonly used clinical treatments because these activities strengthen core muscle functions and promotes recovery of balance function after stroke. Sling exercise training (SET) has been recently emerged as a novel method of training trunk muscles on unstable supporting surfaces. This type of training has been demonstrated to activate trunk muscle activation based upon performance of active exercises with the help of sling exercise equipment. SET concerns the use of a dangling rope and auxiliary equipment to improve physical functions. This type of training has been recently used to facilitate movement rehabilitation after stroke. SET is a safe and partial body weight supporting training. SET could stimulate more proprioceptors , nerve roots, motor organs of the cerebrum and reactivate the muscles. Therefore, the SET could maximizing the sense of balance and enhancing trunk stabilization compared with traditional treatments. SET exhibits better therapeutic effects than traditional treatments, such as Bobath ball sports training and mat exercises.  SET using fixed sling can provide a stable support surface for static balance training, but its length, height and elasticity can also be adjusted for dynamic balance training. Adjustment can be done by setting different moving points and sling power to increase the difficulty of movement, trunk muscle strength, and endurance; moreover, coordination in balance training can be based on the overall improvement of trunk muscle function and increased proprioceptive input, thereby promoting posture control and balance after stroke. | Manuscript2/6 |
| Objectives | 4 | This study aims to evaluate the effect of sling exercise training (SET) on balance in patients with stroke. | Manuscript1 |
| **METHODS** | | |  |
| Protocol and registration | 5 | None. | None |
| Eligibility criteria | 6 | All RCTs on the effect of the SET treatment compared with conventional rehabilitation treatments published in Chinese or English were included in this analysis. The difference in each study, such as observation time points, patients with motor dysfunction, etc. | Manuscript3 |
| Information sources | 7 | We performed a systematic search of literature published prior to October 2, 2015 in PubMed, Cochrane Library, Ovid LWW, CBM (Chinese Biomedical), CNKI (China National Knowledge Infrastructure), WanFang, and VIP databases. | Manuscript3 |
| Search | 8 | Pubmed : (((stroke OR strokes OR stroke patient OR cerebrovascular disorders OR brain injuries OR brain injury, chronic OR paresis OR hemiplegia OR poststroke OR infarction OR thrombus OR Embolus cerebral OR brain OR haemorrhage OR hemorrhage OR haematoma OR hematoma OR bleed)) AND (suspension-assisted training OR suspension-assisted OR suspension OR sling exercise OR sling OR sling exercise training)) AND (balance OR equilibrium OR musculoskeletal equilibrium OR musculoskeletal posture).  VIP databases：M=(核心力量+核心肌群+腹横肌+多裂肌+腹內斜肌+腹外斜肌+腹直肌+腰方肌+背部伸肌群+臀肌+旋髋肌+股后肌群+竖脊肌+盆底肌群+腰背肌+躯干肌+弹力带+悬吊+瑞士球+平衡球+平衡板+悬吊绳+力量练习器械+体能训练)*M= (平衡功能+平衡+协调+协调运动+步态+步态分析+步行+功能独立性测量+足底压力系统+动态肌电图+足印分析法+吸水纸法+鞋跟绑缚标记笔法+躯干控制性+神经肌肉控制)*M=(中风+脑血管病+脑血管病变+脑血管疾病+脑血管畸形+脑动脉瘤+脑血管瘤+脑动静脉畸形+脑血管痉挛+脑血管意外+脑溢血+血管性痴呆+卒中+脑卒中+大厥)。 | Manuscript3 |
| Study selection | 9 | All RCTs on the effect of the SET treatment compared with conventional rehabilitation treatments published in Chinese or English were included in this analysis. The experimental group with the SET treatment with sling devices, device model, treatment of postures, movements, treatment time, intensity, frequency, and treatment courses were unlimited. Except for training trunk muscles, the conventional rehabilitation treatments of the experimental and control groups were unlimited. | Manuscript3 |
| Data collection process | 10 | Two investigators (Chen Lianghua and Chen Jingjie) independently reviewed and extracted information from all eligible publications in accordance with the inclusion and exclusion criteria. Disagreement was resolved by discussion between the two authors. When a consensus was not reached, a third author (Peng Qiyuan) was consulted and a final decision was determined. Data extracted from the publications included the first author, year of publication, study design, number of participants (experimental/control group), interventions for both experimental and control groups (type and duration), outcome measures, the modified Jadad scale score, etc. The methodological quality of each eligible study was independently assessed by two reviewers (Chen Lianghua and Chen Jingjie) based on the modified Jadad scale by using the following criteria. Discrepancies between two independent evaluations for potential articles were resolved by Chen Junqi. | Manuscript3/4 |
| Data items | 11 | Berg balance Scale (BBS), Barthel index score (BI), and Fugl-Meyer Assessment (FMA) were used as independent parameters for evaluating balance function , activities of daily living(ADL) and motor function after stroke respectively, and were subjected to meta-analysis by RevMan5.3 software.  Funding: This work was supported by Tianhe District Science and Technology Projects of Guangdong, China (No.201404KW021). | Manuscript1 |
| Risk of bias in individual studies | 12 | Results were expressed as odds ratio (OR) for dichotomous outcomes or weighted mean difference (WMD)/Standardized Mean Different (SMD) for continuous outcomes with 95% confidence intervals (CI).WMD is used when the units or measurement methods of the same intervention effect are identical. SMD is used when the effect of the same units or interventions are measured by different methods as well as the extremely large mean difference. Heterogeneity may be caused by the difference of intervention time, gender, geographical, the types of research, etc. We used the chi-square-based Q statistic (with a level of significance of P= 0.1) to evaluate the degree of heterogeneity between studies and quantified its extent with the I2 statistic. For P > 0.1 or I2 < 50%, the included studies were identified as having acceptable heterogeneity and the Fixed-effect model was used; otherwise, the random-effects model was used. Sensitivity analysis was performed and meta analysis was repeated to exclude abnormal results if the significant heterogeneity is available (P ≤ 0.1 and I2＞50%). Then assess the stability of the integrative results between the two analysis. P value ≤ 0.1 was regarded as stable when comparing the two analysis. Risk of bias summary (Figure 2). | Manuscript4/5/  Figure 2 |
| Summary measures | 13 | Berg balance Scale (BBS), Barthel index score (BI), Fugl–Meyer movement function score (FMA), sway area (SA) and sway length (SL), BioRescue measures , etc. | Manuscript  3 |
| Synthesis of results | 14 | Berg balance Scale (BBS), Barthel index score (BI), and Fugl-Meyer Assessment (FMA) were used as independent parameters for evaluating balance function , activities of daily living(ADL) and motor function after stroke respectively, and were subjected to meta-analysis by RevMan5.3 software. | Manuscript1 |

Page 1 of 2

| **Section/topic** | **#** | **Checklist item** | **Reported on page #** |
| --- | --- | --- | --- |
| Risk of bias across studies | 15 | The modified Jadad scale by using the following criteria: (1) randomization procedure, (2) allocation concealment, (3) blinding procedure, and (4) drop-out explanation. Aggregate scores ranged from 0 to 7 points. | Manuscript4 |
| Additional analyses | 16 | For P > 0.1 or I2 < 50%, the included studies were identified as having acceptable heterogeneity and the Fixed-effect model was used; otherwise, the random-effects model was used. Sensitivity analysis was performed and meta analysis was repeated to exclude abnormal results if the significant heterogeneity is available (P ≤ 0.1 and I2＞50%). Then assess the stability of the integrative results between the two analysis. P value ≤ 0.1 was regarded as stable when comparing the two analysis. A combined meta-analysis cannot be performed because of the limited number of included studies and different evaluation methods employed. As such, we performed descriptive analysis Yang Guoliang et al. showed that 6 m walking speed and PASS significantly improved after SET. Sun zengxin et al. showed that TIS, 10 m walking speed, extending forward from the upper limbs, and standing time of one suffering leg were significantly improved after SET. Cai chen et al. showed that 10 m walking speed and Holden were significantly improved after SET. Gu shaohua et al. showed that 10 m walking speed and FAC significantly changed after SET. Park, J.H.et al. showed that BioRescue measures such as sway area (SA) and sway length (SL) significantly changed after SET and were similar to those of maximum training performed on the unstable support surface. Lee, J.S. et al. showed that FICSIT-4, TUG, BioRescue score, and EMG significantly changed after SET but were not different from those of maximum training on the unstable support surface. | Manuscript5/6 |
| RESULTS | | |  |
| Study selection | 17 | We initially retrieved 954 articles from the databases that were relevant to the search terms (Figure 1). Nine studies were included for randomized controlled trials. The participants had to be confirmed through head CT or MRI diagnoses and they should be able to understand instructions. | Manuscript4/5  Figure 1 |
| Study characteristics | 18 | Characteristics of the included studies (Table 1). | Table 1 |
| Risk of bias within studies | 19 | Risk of bias summary (Figure 2). | Figure 2 |
| Results of individual studies | 20 | Characteristics of the included studies (Table 1). J.H.et al. and J.S. et al. reported similar effect between SET and traditional treatments, others studies reported SET exhibits better therapeutic effects than traditional treatments. All studies confidence intervals are 95%. | Manuscript1  Table 1 |
| Synthesis of results | 21 | Results of meta-analysis showed that the SET treatment combined with conventional rehabilitation was superior to conventional rehabilitation treatments, with increased degrees of BBS (WMD = 3.81, 95% CI [0.15, 7.48], P = 0.04), BI (WMD = 12.98, 95% CI [8.39, 17.56], P < 0.00001), and FMA (SMD =0.76, 95% CI [0.41, 1.11], P < 0.0001). Based on limited evidence from 9 trials, the SET treatment combined with conventional rehabilitation was superior to conventional rehabilitation treatments, with increased degrees of BBS, BI and FMA, So the SET treatment can be recommended for improvement of balance function after stroke, but the interpretation of our findings is required to be made with caution due to limitations in included trials such as small sample sizes and the risk of bias. | Manuscript1 |
| Risk of bias across studies | 22 | The modified Jadad scale by using the following criteria: (1) randomization procedure, (2) allocation concealment, (3) blinding procedure, and (4) drop-out explanation. Aggregate scores ranged from 0 to 7 points. | Manuscript4 |
| Additional analysis | 23 | For P > 0.1 or I2 < 50%, the included studies were identified as having acceptable heterogeneity and the Fixed-effect model was used; otherwise, the random-effects model was used. Sensitivity analysis was performed and meta analysis was repeated to exclude abnormal results if the significant heterogeneity is available (P ≤ 0.1 and I2＞50%). Then assess the stability of the integrative results between the two analysis. P value ≤ 0.1 was regarded as stable when comparing the two analysis. A combined meta-analysis cannot be performed because of the limited number of included studies and different evaluation methods employed. As such, we performed descriptive analysis Yang Guoliang et al. showed that 6 m walking speed and PASS significantly improved after SET. Sun zengxin et al. showed that TIS, 10 m walking speed, extending forward from the upper limbs, and standing time of one suffering leg were significantly improved after SET. Cai chen et al. showed that 10 m walking speed and Holden were significantly improved after SET. Gu shaohua et al. showed that 10 m walking speed and FAC significantly changed after SET. Park, J.H.et al. showed that BioRescue measures such as sway area (SA) and sway length (SL) significantly changed after SET and were similar to those of maximum training performed on the unstable support surface. Lee, J.S. et al. showed that FICSIT-4, TUG, BioRescue score, and EMG significantly changed after SET but were not different from those of maximum training on the unstable support surface. | Manuscript5/6 |
| **DISCUSSION** | | |  |
| Summary of evidence | 24 | Results of meta-analysis showed that the SET treatment combined with conventional rehabilitation was superior to conventional rehabilitation treatments, with increased degrees of BBS (WMD = 3.81, 95% CI [0.15, 7.48], P = 0.04), BI (WMD = 12.98, 95% CI [8.39, 17.56], P < 0.00001), and FMA (SMD =0.76, 95% CI [0.41, 1.11], P < 0.0001). Based on limited evidence from 9 trials, the SET treatment combined with conventional rehabilitation was superior to conventional rehabilitation treatments, with increased degrees of BBS, BI and FMA, So the SET treatment can be recommended for improvement of balance function after stroke, but the interpretation of our findings is required to be made with caution due to limitations in included trials such as small sample sizes and the risk of bias. | Manuscript5 |
| Limitations | 25 | We included nine studies and the test methods described were different. The varied randomization procedures were not perfect, parts of tests did not specify the kinds of randomization methods, and SET is difficult blinding of patients and doctors, resulting in bias. These may affect test results and high-quality filter paper documents are difficult to obtain. The included studies cover only small sample sizes. Moreover, most trials did not provide drop-out explanations and only partial follow-up tests were carried out to assess the therapeutic effect. Most studies found no consistent effect score, and evaluation was also different, making it is difficult to generalize and obtain authentic meta-analysis results. To obtain more accurate results, larger samples are needed for the multi-center prospective trials and high-quality randomized trials. | Manuscript6 |
| Conclusions | 26 | Based on limited evidence from 9 trials, the SET treatment combined with conventional rehabilitation was superior to conventional rehabilitation treatments, with increased degrees of BBS, BI and FMA, So the SET treatment can improvement of balance function after stroke, but the interpretation of our findings is required to be made with caution due to limitations in included trials such as small sample sizes and the risk of bias. Therefore, more multi-center and large-sampled randomized controlled trials are needed to confirm its clinical applications. | Manuscript7 |
| **FUNDING** | | |  |
| Funding | 27 | The SET treatment can be recommended for improvement of balance, but more multi-center and large-sampled randomized controlled trials are needed to confirm its clinical applications. | Manuscript1 |

Page 2 of 2
